# Assessing the prognostic factors, survival, and recurrence incidence of triple negative breast cancer patients, a single center study in Iran

**DOI:** 10.1371/journal.pone.0208701

**Published:** 2019-01-04

**Authors:** Seied Asadollah Mousavi, Amir Kasaeian, Maziar Pourkasmaee, Ardeshir Ghavamzadeh, Kamran Alimoghaddam, Mohammad Vaezi, Hosein Kamranzadeh Fumani, Davoud Babakhani, Sahar Tavakoli

**Affiliations:** 1 Hematology-Oncology and Stem Cell Transplantation Research Center, Tehran University of Medical Sciences, Tehran, Iran; 2 Deptartment of Biostatistics and Epidemiology, Hematology-Oncology and Stem Cell Transplantation Research Center, Tehran University of Medical Sciences, Tehran, Iran; Fondazione IRCCS Istituto Nazionale dei Tumori, ITALY

## Abstract

**Background:**

Breast cancer is the second leading cause of death due to cancer in women. Triple negative breast cancer (TNBC) is a subgroup with unique behavior. There is a controversy in organ involvement in metastasis. In this study, we planned to define the prognostic factors, survival, and recurrence incidence of patients.

**Materials and method:**

Among the 583 patients with breast mass referred to hematology and oncology clinic in Shariati hospital, Tehran, Iran from March 2005 to March 2015, fifty four patients entered the survival analysis whom we followed for two years until March 2017. Overall survival (OS) and disease-free survival (DFS) and Cumulative recurrence incidences (RI) were estimated. Univariate and multivariate Cox proportional hazards regression was performed to assess risk factors in predicting OS and DFS.

**Results:**

Median follow up for the patients was 5.00 years. The five-year OS, DFS and RI were 86.13% (95% CI (71.42–93.59), 63.09% (95% CI (47.04–75.49) and 32.15% (95% CI (19.52–47.43) respectively. Among the factors studied OS, DFS and RI differed significantly only between patients with and without nodal involvement (P = 0.004, P = 0.003, and P = 0.02 respectively). On the other hand, based on the univariate modeling, patients with nodal involvement had a higher risk of breast cancer-specific death (HR: 17.99, P = 0.004). Furthermore, patients with nodal involvement had a higher risk of breast cancer-specific death or recurrence (HR = 5.64, P = 0.008). In Multivariate model, just the nodal involvement significantly changed the hazard for OS (HR = 23.91, P = 0.001). As the nodal involvement was the only significant risk factor at the 0.2 level of significance, we can consider the hazard ratio of lymph node positivity in DFS univariate models as adjusted hazard.

**Conclusion:**

The only factor with significant effect on OS, DFS and RI was nodal involvement in the pathology report.

## Introduction

Breast cancer is the most prevalent cancer worldwide [1] and TNBC is a subgroup with a distinct clinical and biological characteristic. TNBC can be subdivided according to histopathological features and/or gene expression [2–5]. About 80 percent of TNBC has basal-like subtype identified by using DNA microarray analysis [4, 6]. It is estimated that 25% of TNBCs carry a BRCA1 mutation, and more than 75% of tumors in women who carry the BRCA gene are of triple-negative and/or basal-like phenotype [7]. There are several risk factors for TNBC as BRCA mutation, ethnicity [8, 9], age [6, 8] and Body Mass Index (BMI) [8, 10]. There are no targeted chemotherapy until now for TNBC and the only proven method for systemic management of triple-negative breast cancer for both early-stage and metastatic settings is cytotoxic chemotherapy [11]. Although a first good response to chemotherapy, they have lower OS (overall survival) and DFS (disease free survival) [12–15]. It is presumed that the residual tumor after chemotherapy may relapse soon and cause a poorer outcome [16]. Distant metastasis will peak after three years of diagnosis and decline rapidly [17]. Distant metastasis to lungs and brain are prevalent but bone involvement is rare [18–21].

In Iran, there are limited studies about TNBC. It seems that most clinical and pathological TNBC characteristics in Iranian patients are consistent with other findings in literature, such as younger age at diagnosis, high grade tumors, advanced stage at diagnosis, and short time of 5-year DFS and 5-year OS [22]. Although in some articles bone involvement was rare in the Iranian TNBC patients [23], in the others bone metastasis was more common like other sites of involvement [24]. We tried to study the prognostic factors, the therapeutic approach, and their impact on OS and DFS in the TNBC patients.

## Material and methods

The ethical committee of Tehran University of Medical Sciences approved the study (reference IR.TUMS.REC.1394.1456) and waived the requirement for informed consent. From March 2005 to March 2015, we reviewed the files of all patients in the Hematology and Oncology clinic of Hematology-Oncology and Stem Cell Transplantation Research Center in Shariati hospital, Tehran, Iran. All of the data were fully anonymized in our data bank. Among the patients with breast mass, we found TNBC group and followed them for two years until March 2017. The files were reviewed for the first and the last visit, age, laterality, size, lymph node involvement, stage of tumors, perineural, vascular and marginal invasion, P53 mutation, Ki67 expression, BRCA mutation, BMI, type of surgery, chemotherapy regimen, radiotherapy, time of recurrence and the organs involved when recurrence occurred.

To define estrogen, progesterone and human epidermal growth factor receptor positivity we checked the pathology report. If ER and PR were more than one percent (> 1%), we assumed them positive. For HER2 receptor, we considered zero and 1+ negative and 3+ positive by Immunohistochemistry (IHC). For HER2 2+ in IHC we did a further complementary FISH (Fluorescence In Situ Hybridization) to detect the true positive results. We defined TNBC as having all three receptors (ER, PR, HER2) negative. No test was done on CK5/6 or EGFR (HER1). For Ki67 expression and P53 mutations, we checked the IHC report. While the prognostic value of Ki-67 in TNBC remains to be determined we assumed the expression of Ki67 equal to or more than 20 percent positive, according to the Thirty-Eighth Annual CTRC-AACR San Antonio Breast Cancer Symposium [25]. Any positive report for P53 was considered positive.

Because we did not have the patients’ time of menopause, we made a division by the age of 50 to show the prevalence of TNBC in accordance with this age. We used the American Joint Committee on cancer (AJCC) staging by TNM system to define the stage of the tumors. Lymph node status was described according to the number of regional lymph nodes with pathologically proven metastasis. Distant metastasis were proved in the patients by complementary imaging like CT scan, whole body bone scan, MRI and sonography and if accessible, biopsies were taken. The type of surgery categorized the patients into two groups; those who underwent MRM (modified radical mastectomy) and those with BCT (breast conservative therapy). Because of diversity in the regimens of chemotherapy, we divided the patients into three groups: 1) those who received cyclophosphamide and anthracycline (Doxorubicin or Epirubicin) and continued with Docetaxel (AC-T or EC-T), 2) those who received Docetaxel, Doxorubicin and Cyclophosphamide (TAC) and 3) those who underwent other regimens consists 5-FU (Fluorouracil). These are 5-FU plus cyclophosphamide and a third drug like anthracycline (Doxorubicin or Epirubicin) or Methotrexate (CMF).

We updated the data and followed the patients for two years until March 2017, and reviewed the protocol by our team of ethics. The ethics committee waived the requirement for informed consent. All of the data are fully anonymized in our data bank.

Overall survival (OS) was measured from the time of diagnosis to the time of death, or the last contact in surviving patients. Disease free survival (DFS) was defined as the time between the diagnosis and the recurrence, death or to the last contact in patients without recurrence or death event. Recurrence was defined as coming back of breast cancer in the local site (e.g. in the treated breast or near the mastectomy scar) or somewhere else in the body. Imaging and biopsies were done to prove the diagnosis of recurrence.

Kaplan–Meier curves were derived to determine overall survival and disease-free survival, and were compared by means of the log-rank test. Median follow-up time was established with the reverse Kaplan-Meier method. The assumption of proportionality of hazards was checked using Schoenfeld residuals (results not shown).

Cumulative incidences analysis was used to estimate recurrence incidence in each group, which were compared via Gray's test.

Univariate and multivariate analyses were performed using a Cox proportional hazard regression model for survival. Because there was significant censoring, Firth’s bias correction procedure through penalized likelihood methods was used to calculate hazard ratios (HRs) and 95%confidence intervals. All the variables with a P-value at or below 0.2 in the univariate analysis were included in the multivariate analysis. Analyses were done with STATA version 11.2 and Packages "survival", "cmprsk", and "coxphf" in R software version 3.3.1.

## Results

**Fig 1:**
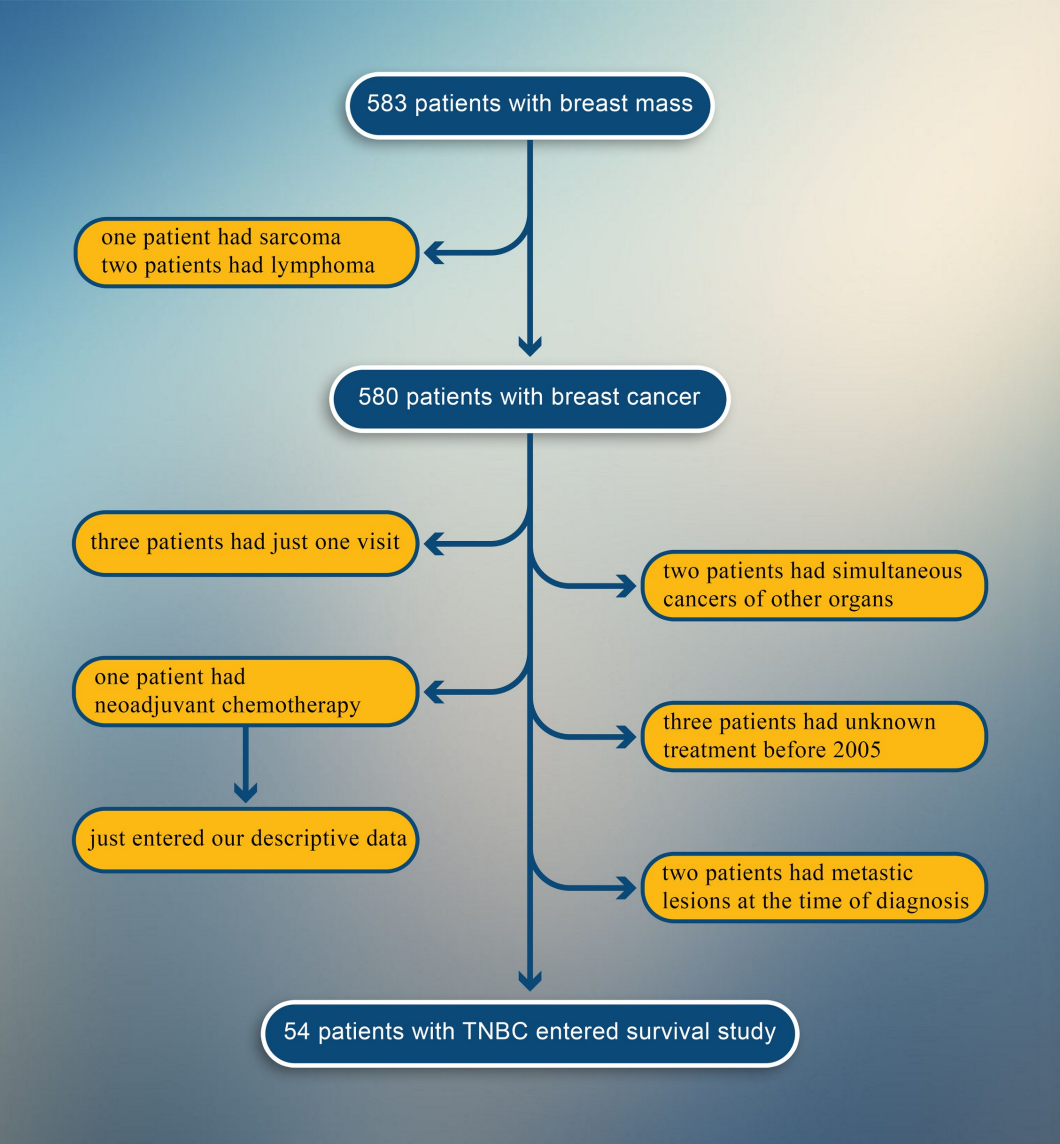
Algorithm that shows the selection path of TNBC patients.

Among 583 patients came to the Hematology-Oncology clinic with breast mass from March 2005 to March 2015, one patient had sarcoma and two of them had lymphoma. Only 512 patients with breast cancer had defined all of their receptor status. Sixty five patients had triple negative breast cancer (TNBC). Thus, the prevalence of TNBC in our study was about 12.69 percent. Only two of them had checked BRCA mutations, which one of them was positive for BRCA 1 & 2. In the end, 54 patients entered our study for analysis (Fig 1).

In the files we reviewed, we could define just 29 patients for perineural invasion that four of them (14 percent) was positive. Again, 13 (36%) out of 36 patients had vascular invasion and three (9%) out of 34 patients had the margins involved in pathology report. Ki67 expression had been defined in only eighteen patients (minimum: 0 maximum: 90%). We reviewed the files again to be sure about them and analyzed the remaining data. Table 1 shows the variables we reviewed in this study.

**Table 1:** Variables studied in the TNBC patients.

variable	division	Number (%) Total: 54 (100)
**laterality**		
	right	22 (40.74)
	left	32 (59.25)
**stage**		
	I	5 (9.26)
	IIA	23 (42.59)
	IIB	14 (25.92)
	IIIA	4 (7.40)
	NA	8 (14.81)
**Tumor size**		
	1 cm< *T1C* <2 cm	11 (20.37)
	2 cm< *T2* <5 cm	31 (57.40)
	5 cm< T3	6 (11.11)
	NA	6 (11.11)
**BMI**		
	<25	11 (20.37)
	25≤BMI	19 (35.18)
	NA	24 (44.44)
**Age**		
	≤ 50	36 (66.67)
	50 <	18 (33.33)
**Node involvement**		
	Positive	23 (42.59)
	negative	27 (50)
	NA	4 (7.40)
**Ki67 expression**		
	20 ≤	12 (22.22)
	10 ≤ ki67 < 20	3 (5.55)
	<10	3 (5.55)
	NA	39 (72.22)
**P53 mutation**		
	positive	20 (37.03)
	negative	12 (22.22)
	NA	22 (40.54)
**Chemotherapy regiment**		
	AC-T or EC-T	32 (59.26)
	CAF, CEF, CMF	15 (27.77)
	TAC	3 (5.55)
	NA	4 (7.41)
**Type of surgery**		
	MRM	41 (75.92)
	BCT	11 (20.37)
	NA	2 (3.70)
**Radiotherapy**		
	Without	15 (27.77)
	With	30 (55.55)
	NA	9 (16.67)

NA: Not Available

We found that during the follow up 16 out of 54 patients had recurrence. Local recurrence was the most prevalent (7 out of 16) and lungs (5 out of 16), bone (4 out of 16), and liver (3 out of 16) were among the prevalent distal organ involvement. We had four patients with bone metastasis with simultaneous lung involvement in two of them. There was only one patient with brain metastasis with lung and bone involvement at the same time. A carcinoid tumor of the lung found in a patient who had a liver metastasis of her breast cancer simultaneously that confirmed by biopsy. Table 2 shows the metastasis locations in the TNBC patients during follow up.

**Table 2:** Metastasis locations in the TNBC patients during follow up.

Distal Metastasis Site	Number of Subjects	
	N: 54	percent
adenopathy	2	3.70
local	7	12.96
bone	4	7.40
lungs	5	9.26
liver	3	5.55
brain	1	1.85
Bone + Lungs + Brain	1	1.85
Bone + Lungs + Liver	1	1.85
Local + Lungs	1	1.85
Adenopathy + Bones	1	1.85
Liver + Carcinoid	1	1.85
Total Recurrence	16	29.63

**Fig 2:**
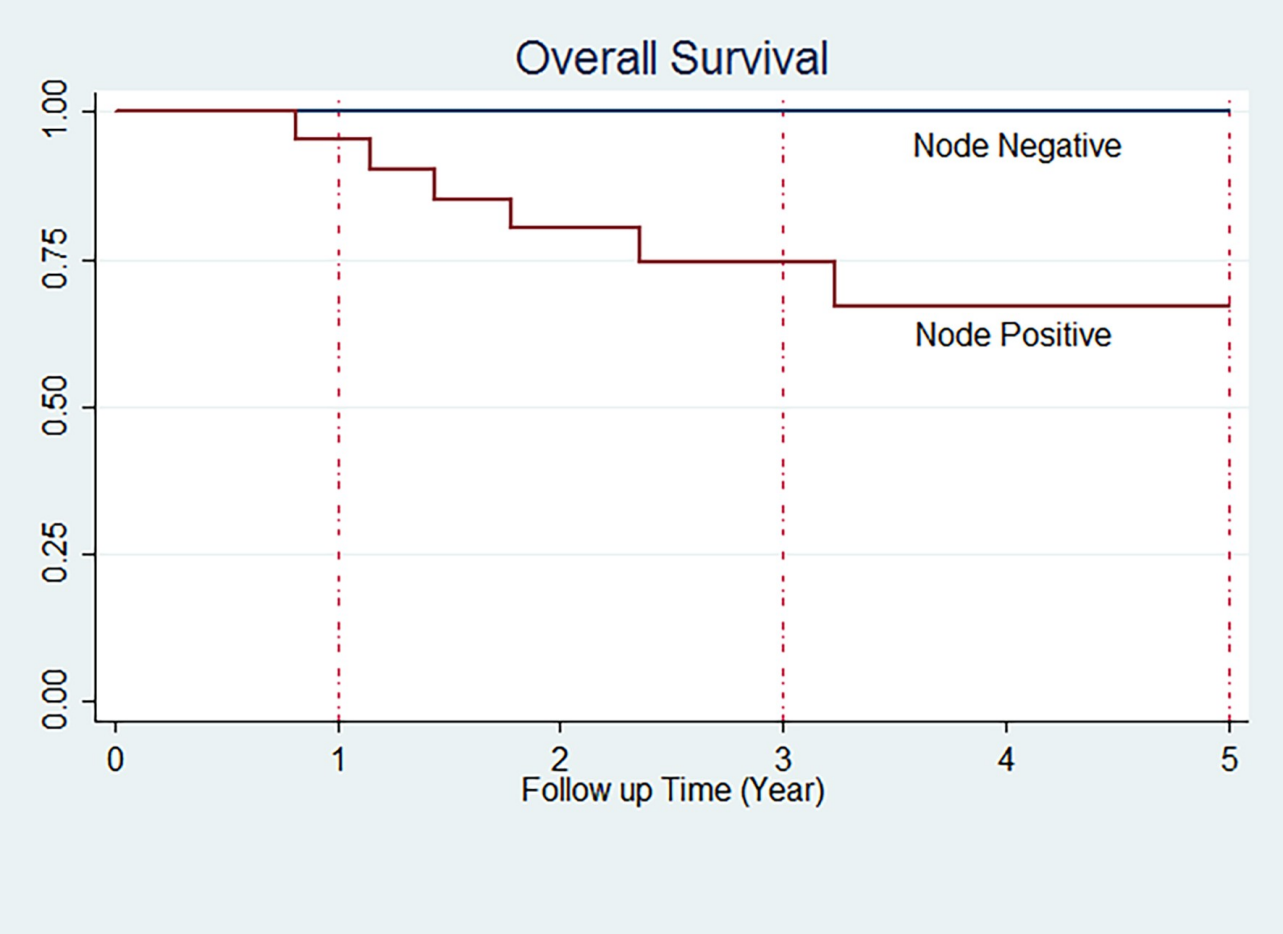
Overall survival of TNBC patients in node positive and node negative groups.

Mean age was 45.75 and median age was 46 years. Median follow up for them was 5.00 years. Five-year OS was 86.13 (95% CI: 71.42–93.59). Three year and five year OS of patients according to the variable are shown in Table 3. In our study, nodal involvement significantly changes the OS (P value: 0.004) (Fig 2). We found no significant difference in OS according to other variables.

**Table 3:** Three and Five year OS, DFS and recurrence of the patients according to the variables.

variable	division	3 year OS% (CI95%)	5 year OS% (CI95%)	P Value ∫	3 year DFS% (CI95%)	5 year DFS% (CI95%)	P Value ∫	3 year recurrence% (CI95%)	5 year recurrence% (CI95%)	P Value ∫∫
**laterality**	left	81.72 (61.27–92.02)	81.72 (61.27–92.02)	0.18	72.59 (52.31–85.35)	62.57 (40.68–78.32)	0.709	20.4 (8.06–36.68)	31.16 (13.71–50.44)	0.869
	right	100	93.33 (61.26–99.03)		70.47 (45.66–85.53)	64.60 (39.48–81.43)		29.25 (11.46–49.77)	35.14 (14.95–56.25)	
**stage**	I	100	100	-	100	100	-	0	0	-
	IIA	94.74 (68.12–96.24)	88.20 (60.13–96.95)	0.366	80.20 (55.45–92.08)	66.24 (38.57–83.68)	0.580	15.37 (3.59–34.84)	29.85 (9.80–53.29)	0.172
	IIB	82.59 (45.87–95.41)	82.59 (45.87–95.41)		66.67 (32.30–86.46)	66.67 (32.30–86.46)		25.93 (5.04–54.34)	25.93 (5.04–54.34)	
	IIIA	71.43 (8.97–95.41)	-	-	45 (3.30–82.95)	-	-	50 (2.25–88.09)	-	-
**Size**	≤5 cm	91.49 (75.80–97.18)	87.97 (70.81–95.35)	0.313	73.24 (55.87–84.65)	66.37 (48.04–79.51)	0.388	21.6 (9.95–36.14)	28.55 (14.44–44.39)	0.261
	5 cm <	77.78 (16.64–96.54)	-		59.52 (10.91–88.56)	-		44.44 (1.61–85.26)	-	
**Age**	≤50 yo	92.30 (72.38–98.03)	92.30 (72.38–98.03)	0.132	71.19 (51.67–83.95)	62.81 (42.41–77.72)	0.912	25.22 (11.61–41.42)	34.12 (17.13–51.92)	0.748
	50 yo <	83 (56.03–94.18)	76.08 (47.79–90.37)		71.43 (44.30–87.02)	63.03 (34.50–81.85)		23.01 (6.73–44.94)	30.95 (10.22–54.71)	
**BMI**	< 25	90 (47.30–98.53)	90 (47.30–98.53)	0.146	60.71 (25.96–83.14)	60.71 (25.96–83.14)	0.404	10 (0.45–37.41)	30 (6.13–59.49)	0.834
	25 ≤	100	100		83.04 (55.97–94.22)	74.30 (43.76–89.87)		17.03 (3.94–37.94)	25.33 (6.97–49.28)	
**Nodal** **involvement**	Negative	100	100	***0*.*004***	95.65 (72.93–99.38)	83.36 (56.00–94.45)	***0*.*003***	4.17 (0.28–17.99)	16.94 (3.75–38.29)	***0*.*02***
	Positive	74.64 (49.15–88.65)	67.73 (40.69–84.25)		51.31 (28.07–70.44)	45.28 (22.77–65.42)		39.35 (17.90–60.31)	45.71 (21.93–66.78)	
**Ki67 expression**	<20%	83.33 (27.31–97.47)	83.33 (27.31–97.47)	0.91	83.33 (27.31–97.47)	83.33 (27.31–97.47)	0.306	0	0	0.143
	20% ≤	73.53 (28.34–92.78)	73.53 (28.34–92.78)		53.97 (17.76–80.30)	53.97 (17.76–80.30)		35.71 (4.86–70.42)	35.71 (4.86–70.42)	
**P53 mutation**	negative	100	100	0.433	74.21 (39.43–90.88)	74.21 (39.43–90.88)	0.679	26.66 (5.65–54.43)	26.66 (5.65–54.43)	0.907
	positive	92.59 (57.89–98.92)	92.59 (57.89–98.92)		68.76 (40.04–85.79)	60.67 (31.77–80.43)		24.10 (6.89–46.91)	32.73 (10.52–57.46)	
**chemotherapy**	AC-T or EC-T	88.64 (68.61–96.21)	83.26 (60.63–93.52)	0.151	71.79 (51.19–84.87)	56.00 (33.41–73.59)	0.251	24.91 (10.58–42.31)	41.36 (20.38–61.27)	0.336
	5FU based	100	100		79.22 (48.37–92.80)	79.22 (48.37–92.80)		20.55 (4.61–44.32)	20.55 (4.61–44.32)	
**surgery**	BCT	85.71 (33.41–97.86)	85.71 (33.41–97.86)	0.877	66.67 (27.17–88.15)	66.67 (27.17–88.15)	0.925	20 (2.64–49.03)	20 (2.64–49.03)	0.721
	MRM	88.71 (72.60–95.62)	84.94 (67.18–93.52)		69.89 (52.09–82.14)	62.94 (44.45–76.77)		27.49 (14.02–42.79)	34.33 (18.83–50.42)	
**radiotherapy**	Without	92.59 (57.89–98.92)	92.59 (57.89–98.92)	0.368	79.02 (48.05–92.72)	69.73 (37.28–87.65)	0.361	20.55 (4.61–44.32)	29.38 (8.10–55.04)	0.489
	With	86.57 (63.66–95.50)	79.65 (53.22–92.12)		60.21 (38.28–76.49)	54.48 (32.21–72.20)		36.28 (17.44–55.50)	42.21 (21.03–62.06)	

∫ The data were analyzed by log rank test

∫∫ The data were analyzed by Gray test

**Fig 3:**
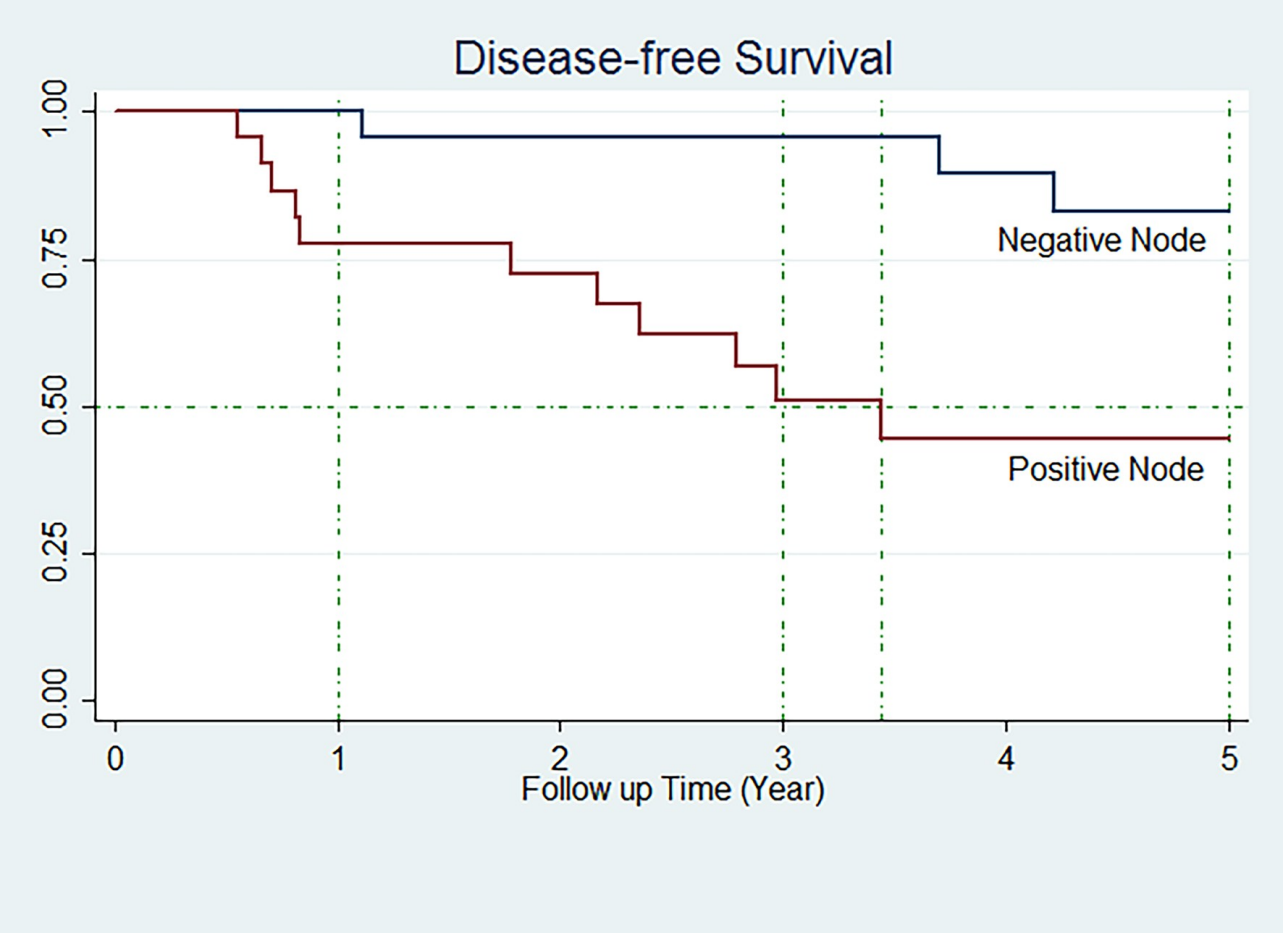
DFS of TNBC patients in node positive and node negative groups.

Three and five year DFS were 71.28 (95% CI: 56.22–81.95) and 63.09 (95% CI: 47.04–75.49) respectively. DFS of patients according to the variables is shown in Table 3. DFS changes significantly when nodal involvement exists (P Value: 0.003) (Fig 3). There is no significant difference in DFS according to other variables.

**Fig 4:**
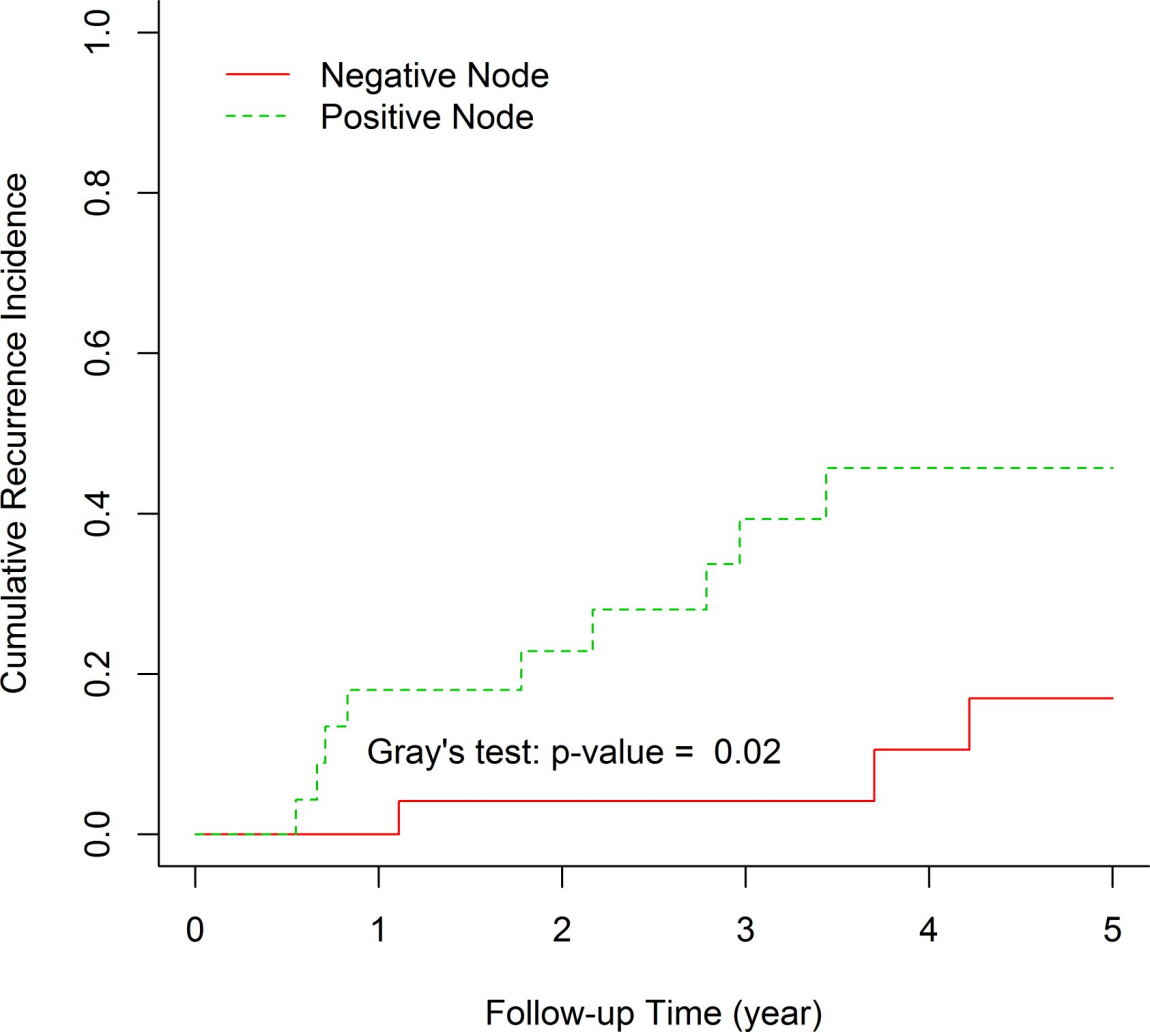
Recurrence in TNBC patients in node positive and node negative groups.

Five-year recurrence incidence (RI) after treatment was 33.15 (95% CI: 19.52–47.43). Table 3 shows the recurrence incidence of TNBC after treatment according to the variables. We found that nodal involvement will change RI significantly (P value: 0.02) (Fig 4) but other variables did not. The five-year RI after treatment in node positive patients was 45.6 percent (95% CI: 21.93–66.78) in contrast to 16.94% (95% CI: 3.75–38.29) in node negatives. Although insignificant (P: 0.336), the standard chemotherapy AC-T or EC-T has higher recurrence (41.36%) after 5 years (95% CI: 20.38–61.27).

We estimated the hazard ratios for OS and DFS for the TNBC patients that are shown in Table 4. BMI, Nodal involvement, P53 mutation and chemotherapy regimens were the four factors that HRs were estimated based on Firth method to correct the significant censoring bias. Nodal involvement positivity significantly changed the results for OS (HR: 17.998, P value: 0.004) and DFS (HR: 5.64, P value: 0.008). In this study some other factors (although insignificant) decrease the hazard ratio in OS and DFS. HR of patients with BMI ≥ 25 is 0.16. This means that patients with overweight or obesity decreases the hazard of death about 84 percent (P: 0.214). In addition, right side breast cancer and 5-FU based chemotherapy decreases the hazard of death about 74 percent (HR: 0.26, P: 0.217) and 78 percent (HR: 0.22, P: 0.210) respectively. Similarly, HR for death or recurrence decreases for BMI ≥ 25 (HR: 0.56, P: 0.41), right side breast cancer (HR: 0.83, P: 0.71) and 5-FU based chemotherapy (HR: 0.48, P: 0.26).

**Table 4:** Hazard Ratio of the patients for OS and DFS according to the variables.

		Univariate Modeling		Multivariate Modeling			
Variable	Division	Hazard Ratio for OS (CI95%)	P-value	Hazard Ratio for OS (CI95%)	P-value	Hazard Ratio for DFS (CI95%)	P-value
**laterality**	Left						
	right	0.26 (0.3–2.21)	0.217			0.83 (0.31–2.18)	0.710
**Stage**	IIA						
	IIB	2.41 (0.33–17.25)	0.381			1.43 (0.4–5.11)	0.582
**Size**	≤5 cm						
	5 cm <	3.13 (0.30–32.24)	0.337			1.95 (0.41–9.21)	0.396
**Age**	≤ 50 yo						
	50 yo <	3.41 (0.62–18.63)	0.157	4.17 (0.92–23.95)	0.06	0.94 (0.35–2.56)	0.912
**BMI**	< 25						
	25 ≤	0.16^§^ (0.001–2.96)	0.214			0.55 (0.14–2.24)	0.411
**Nodal involvement**	Negative						
	Positive	17.99^§^ (2.11–2350)	***0*.*004***	23.91 (2.74–3139)	***0*.*001***	5.64 (1.56–20.31)	***0*.*008***
**Ki 67**	<20%						
	20% ≤	1.15 (0.10–12.69)	0.909			2.67 (0.29–24.17)	0.381
**P53**	Negative						
	Positive	1.84^§^ (0.09–269.39)	0.694			1.34 (0.33–5.38)	0.680
**Chemotherapy**	AC-T or EC-T						
	5FU based	0.21^§^ (0.001–2.02)	0.210			0.47 (0.13–1.72)	0.261
**Surgery**	BCT						
	MRM	1.18 (0.14–10.14)	0.878			0.94 (0.26–3.31)	0.925
**Radiotherapy**	Without						
	With	2.64 (0.29–23.81)	0.386			1.70 (0.54–5.39)	0.366

§ HRs were estimated based on Firth method to correct the significant censoring bias.

Tumors with positive Ki67 expression increased the HR for OS (HR: 1.15, P: 0.91) and DFS (HR: 2.67, P: 0.38) but this result was insignificant. Again tumors with positive P53 mutation increased the HR for OS (HR: 1.84, P: 0.694) and DFS (HR: 1.34, P: 0.68) but this result was insignificant as well.

Based on the univariate modeling, patients with lymph node involvement had a higher risk of breast cancer-specific death (HR: 17.99, P = 0.004) than those without lymph node involvement. For multivariate model, we chose the variables with the level of significance below 0.2. Thus an analysis done for age and nodal involvement. Based on multivariate model age above 50 years increased the HR 4.17 times but this result was insignificant (P: 0.06). Nodal involvement significantly changed the HR in multivariate analysis (HR: 23, P: 0.001).

Furthermore patients with lymph node involvement had a higher risk of breast cancer-specific death or recurrence (HR = 5.64, P = 0.008) than those without lymph node involvement. As the lymph node involvement positivity was the only significant risk factor at the 0.2 level of significance, we can consider the hazard ratio in the univariate models as adjusted hazard ratios

## Discussion

In our study, we found that about 13 percent of our patients were TNBC defined as ER, PR and HER2 negative. In the international studies TNBC consists about 10 to 20 percent of all breast cancers worldwide and most of the data are in accordance with the prevalence worldwide [23, 24], but some studies show a higher prevalence in India and Ghana [26, 27] and only one study in Iran reported a lower prevalence [28].

It is known that TNBC has a good response to chemotherapy but because of the lack of target therapy in follow up it has poorer prognosis in OS and DFS. While Ovaricek et al [29] showed 74.5 and 68.2 percent for OS and DFS in five years respectively a recent study on invasive TNBC patients by Kimberly Thomas et al [30], in a 5-year follow-up showed 82.8% OS and 77.2% DFS for the invasive TNBC patients. The difference between the two studies can be due to a difference in ethnicity and the available health care system for the patients. It is mentioned in the latter study that African-American (AA) women had significantly lower OS compared with Caucasian American (CA) women over a 5-year period, 23.4% versus 64.8%, respectively (HR, 2.192; 95% CI 1.058, 4.543, *P* = .04). AA women also had a significantly longer time delay from diagnosis to treatment compared with CA women (61 versus 43 days respectively, *P* = .005). In a study of Aghili et al in Iran [28, 31] three and five year survival were 69.8 and 62.3 percent respectively. Our study shows 89.05 and 86.13 percent for 3 and 5 year OS, which is comparable with the Kimberly Thomas study. In our patients, we found a 63.09 percent for the 5-year DFS. The lower DFS in our study can be due to a poorer socioeconomic matter, which needs a thorough study.

As mentioned before although in some articles bone involvement was rare in the Iranian TNBC patients [23], in the others bone metastasis was more common like other sites of involvement [24]. We found bone metastasis as prevalent (4 out of 16) as other organs like the lungs (5 out of 16) and liver (3 out of 16). In one patient, bone involvement was the only organ of involvement in recurrence. Local recurrence has the highest prevalence of recurrence in our study (7 out of 16). In a study of Lori M. van Roozendaal et al [32] it is mentioned that Triple-negative clinically T1-2N0 breast cancer patients rarely develop a regional recurrence. In contrast, Wang SL et al [33] found a greater for locoregional recurrence in node positive TNBC patients after mastectomy. It seems that nodal involvement can have a great affect for local and regional involvement after standard appropriate therapy. To show the prevalence of the organ of recurrence needs a perfect study with greater sample size.

Unfortunately we did not have data for BRCA mutations but it is known that it is higher in the younger patients as the prevalence of TNBC is higher in them [34, 35]. We found no significant change in OS or DFS because of P53 mutation and Ki67 expression. Although insignificant, it seems that most factors like P53 mutation and Ki67 expression have affected the OS in the first three years and the survival afterward is fixed. Although they are known to be effective in other studies [36, 37]. It is supposed that higher Ki67 expression in breast tumors will make them resistant to anthracycline [38]. It is possible in a larger sample size we can prove the effect of P53 mutation and Ki67 expression on the survival of TNBC patients.

We designed to study on the number of child delivery, lactation and the age of menopause but the data of these variables were not complete. The effect of BMI on OS and DFS in the previous studies is controversial. Widschwendter et al [39] showed a lower OS and DFS in the patients with BMI more than 40. Ping-Ping Bao et al [40] showed a lower OS and DFS in the patients with BMI higher than 28. But Burcu Cakar et al [41] did not show any significant relationship between BMI and the survival. We did not find a significant relationship between BMI and OS or DFS too. Although BMI can indicate a good nutrition and we know that in some disease like CKD it has paradoxical effect, still it can be studied with a larger sample size to understand the net effect of BMI on survival.

We found no significant change in our study in OS or DFS according to tumor laterality. It seems that left breast tumor is more prevalent than the right although right breast involvement increased the hazard ratio in OS and DFS insignificantly. Zeeneidin AA et al [42] showed a lower survival in left breast tumor but Fatima N et al [43] showed higher invasion characteristics in right breast tumors. In contrast, Bao J et al [44] showed no difference in prognosis between right and left breast tumors.

NPI (Nottingham Prognostic Index) is validated in many studies [45–47]. We did not studied histologic grade but the size of the tumor and nodal involvement were overviewed. The only factor that significantly changed the OS and DFS was nodal involvement in our study (P value: 0.004, 0.003 respectively). We did not see a significant change in OS or DFS because of tumor size in our study. It is maybe due to an insufficient number of patients entered the study.

Many studies report that the stage of tumor is the most significant factor that affect prognosis. We did not have sufficient number of patients in stages I and IIIA. Thus, we studied on the remaining patients in stages IIA and IIB. Although, stage IIB had relatively poorer OS and DFS this difference was not statistically significant.

We found a lower (not significant) OS in the patients above the age 50. Although this result was insignificant. It is maybe due to the existence of other diseases that makes the elders susceptible to death. Although it should be studied separately with sufficient number of patients. DFS and RI did not change significantly according to the age group. Carey K. Anders et al [48] showed that TNBC in patients below the age of 40 were more invasive. Wenji Zhu et al [49] showed that patients above 70 years old had higher mortality and morbidity in the first two years after treatment. Judith April Malmgren et al [50] said that 18 percent of patients above the age 75 will die for reasons other than breast cancer but disease specific survival did not changed significantly. Nilufer et al [51] did not see a significant change in survival after neoadjuvant or adjuvant chemotherapy.

In previous studies no significant difference was seen between the standard chemotherapy regimen (AC-T) and CMF in OS or DFS. In some studies, CMF was superior to the others [52]. Minsung Kim et al [53] showed no significant different between AC-T and 5-FU based chemotherapy in OS, DFS and RI although, it seems that in 5-FU based group the results are better. Mousavi et al [54] studied on 40 patients. 20 of them got CMF and 20 received AC-T standard therapy. No difference found in the two groups. We found the 5-FU based chemotherapy superior in the OS, DFS, and RI, but they were insignificant. We could not study on the patients received TAC regimens because of insufficient number of patients in this group. It is not obvious that the nature of the tumor itself responds better to the regimen or the selection of the patients chosen to receive 5-FU based chemotherapy makes them respond better to this regimen. It seems that to find out the answer needs a perfect study on the chemotherapy regimens.

Laurent T et al [55] declared that radiotherapy was useful in patients underwent BCT surgery but not MRM. But M.A. O,Rorke et al [56] found it useful in reducing recurrence in both BCT and MRM group but they didn’t find it effective on OS. In our study, we did not find radiotherapy significantly effective on OS, DFS or recurrence but it seems that those who underwent radiotherapy had poorer OS and DFS. This effect may be caused by the poor prognosis of the patients per se chosen to receive radiotherapy. For example, the prevalence of nodal involvement and larger tumor size that were higher in our study.

We did not find type of surgery to be effective on prognosis. From 41 patients underwent MRM only four patents had recurrence that three of them had received radiotherapy. From 11 patients who underwent BCT only two of them had recurrence which one of them had not received radiotherapy. We could not document the net effect of the type of surgery on OS, DFS or recurrence because of the low number of patients.

## Conclusion

From the factors we studied on TNBC patients we found no significant change in OS, DFS or RI according to age, laterality, tumor size, stage of tumors, type of surgery, chemotherapy regimen, radiotherapy and BMI. P53 mutation and Ki67 expression were not significantly affect the OS and DFS. The only significant factor that affect OS, DFS and RI was nodal involvement. We found that local and regional involvement was the most prevalent site of recurrence and indeed bone metastasis was among the prevalent organs involved in recurrence. Although NPI is the most significant factor that can affect OS and DFS in the patients, because of insufficient number of patients entered the study, we could not show the net effect of tumor size on the OS or DFS. We found a lower (not significant) OS in the patients above the age 50. In addition, we found the 5-FU based chemotherapy superior in the OS, DFS, and RI, but they were insignificant and needs a further complete study with more meticulous selection criteria. We did not find the type of surgery to be effective on prognosis too.

## Supporting information

S1 Database The least database of the patients that did not need the patients consent. Days to the last visit, to the recurrence date and to the death time was estimated which can be helpful to calculate OS and DFS. The patients marked with yellow color were omitted from the survival study.
(XLSX)
